# Breaking new ground: machine learning enhances survival forecasts in hypercapnic respiratory failure

**DOI:** 10.3389/fmed.2025.1497651

**Published:** 2025-02-20

**Authors:** Zhongxiang Liu, Bingqing Zuo, Jianyang Lin, Zhixiao Sun, Hang Hu, Yuan Yin, Shuanying Yang

**Affiliations:** ^1^Department of Respiratory and Critical Care Medicine, The Second Affiliated Hospital of Xi’an Jiaotong University, Xi’an, Shanxi, China; ^2^Department of Respiratory and Critical Care Medicine, The Yancheng Clinical College of Xuzhou Medical University, The First People’s Hospital of Yancheng, Yancheng, China; ^3^Disease Prevention and Control Center of Funing County, Yancheng, China; ^4^Department of Respiratory and Critical Care Medicine, The First Affiliated Hospital of Nanjing Medical University, The People’s Hospital of Jiangsu Province, Nanjing, China

**Keywords:** hypercapnic respiratory failure, survival model, random survival forest, deep learning-based survival prediction algorithm, Cox proportional risk

## Abstract

**Background:**

The prognostic prediction of patients with hypercapnic respiratory failure holds significant clinical value. The objective of this study was to develop and validate a predictive model for predicting survival in patients with hypercapnic respiratory failure.

**Methods:**

The study enrolled a total of 697 patients with hypercapnic respiratory failure, including 565 patients from the First People’s Hospital of Yancheng in the modeling group and 132 patients from the People’s Hospital of Jiangsu Province in the external validation group. The three selected models were random survival forest (RSF), DeepSurv, a deep learning-based survival prediction algorithm, and Cox Proportional Risk (CoxPH). The model’s predictive performance was evaluated using the C-index and Brier score. Receiver operating characteristic curve (ROC), area under ROC curve (AUC), and decision curve analysis (DCA) were employed to assess the accuracy of predicting the prognosis for survival at 6, 12, 18, and 24 months.

**Results:**

The RSF model (c-index: 0.792) demonstrated superior predictive ability for the prognosis of patients with hypercapnic respiratory failure compared to both the traditional CoxPH model (c-index: 0.699) and DeepSurv model (c-index: 0.618), which was further validated on external datasets. The Brier Score of the RSF model demonstrated superior performance, consistently measuring below 0.25 at the 6-month, 12-month, 18-month, and 24-month intervals. The ROC curve confirmed the superior discrimination of the RSF model, while DCA demonstrated its optimal clinical net benefit in both the modeling group and the external validation group.

**Conclusion:**

The RSF model offered distinct advantages over the CoxPH and DeepSurv models in terms of clinical evaluation and monitoring of patients with hypercapnic respiratory failure.

## Introduction

Hypercapnic respiratory failure (HRF) is typically defined as the presence of elevated arterial carbon dioxide levels (pCO2 > 45 mmHg), often accompanied by hypoxemia (pO2 < 60 mmHg) (1, 2). HRF is commonly associated with respiratory diseases, including chronic obstructive pulmonary disease (COPD), obstructive sleep apnea syndrome/hypopnea syndrome (OSASH), and congestive heart failure (CHF) (3, 4). Each of these ailments can contribute to impaired failure, resulting in a significant number of patients experiencing “multifactorial” HRF. The potential causes of HRF are not readily apparent upon initial assessment, and there may even be concurrent multiple etiologies requiring targeted treatment. Some researchers still regard HRF as a singular yet heterogeneous disease (5).

HRF is prevalent in clinical settings, with the majority of patients necessitating hospitalization, thereby incurring substantial healthcare expenditures (6). Furthermore, HRF is associated with adverse clinical outcomes, and the onset of HRF is predictive of shortened survival time. Specifically, the 1-year, 3-year, and 5-year survival rates were 81, 59, and 45%, respectively, indicating a significantly elevated mortality risk (approximately tenfold) compared to individuals of similar age without this condition (4). Therefore, the factors influencing the outcomes of HRF patients warrant careful consideration.

Currently, there is a paucity of research on the prevalence and prognosis of HRF as an individual entity. Despite excluding underlying diseases and comorbidities, the available study remains relatively limited in scope and fails to address the prediction of mortality risk (7–9). There is currently no existing clinical model available to assess the prognostic survival risk of an individual with HRF.

To more effectively address these issues, numerous experts and clinicians have devoted themselves to ascertain prognostic models for respiratory failure (10, 11). The current prevailing models used for event timing prediction include Cox proportional risk models (commonly known as Cox regression), random survival forests, and DeepSurv (nonlinear versions of Cox regression utilizing deep learning techniques) (12).

However, the limitations of traditional survival prediction tools are primarily manifested in their inadequate handling of nonlinear relationships and complex interactions, limited adaptability to high-dimensional data, challenges in addressing data heterogeneity and missing values, insufficient individualized predictive capabilities, and limited explanatory power (13). Machine learning approaches, such as random survival forests (RSF), effectively address these challenges by leveraging advanced nonlinear modeling, high-dimensional data processing, robust training mechanisms, and interpretable tools. These capabilities offer more flexible and efficient solutions for precision medicine and personalized therapy, thereby demonstrating significant potential in complex clinical settings (14, 15).

Therefore, assessing algorithm accuracy and comparing performance across different prediction algorithms was a crucial aspect of our study due to the intricacy of the data and algorithms involved.

The objective of this study is to comprehensively gather pertinent information on influential factors in hospitalized patients with HRF, conduct a comparative analysis and selection of three predictive models for survival and prognosis, and assess the clinical significance of variables in prediction, thereby providing a practical prognostic prediction tool for managing patients with HRF.

## Materials and methods

### Data sources

The training set comprised a total of 565 patients diagnosed with hypercapnic respiratory failure who were admitted to the First People’s Hospital of Yancheng from October 2020 to September 2021. The external validation dataset consisted of 132 patients with hypercapnic respiratory failure hospitalized at the People’s Hospital of Jiangsu Province between October 2021 and December 2021. The primary objective of this data partitioning was to assess the model’s generalizability across different institutions, patient populations, and time periods. The training dataset included a relatively large number of patients primarily sourced from a regional hospital, while the external validation dataset originated from a higher-level medical institution, thereby simulating the diversity of real-world application scenarios. Although the current datasets are derived from two hospitals, we have selected data from different levels of care and across continuous time periods, which enhances the robustness of the model’s generalization. To demonstrate consistent performance across diverse datasets, we also evaluated the model’s generalizability.

The criteria for research patients with HRF were as follows: Arterial oxygen partial pressure (PaO2) was less than 8.0 kPa (60 mmHg) and arterial carbon dioxide partial pressure (PaCO2) was greater than 6.0 kPa (45 mmHg) based on blood gas analysis (16).

Patients who had incomplete clinical data, were under the age of 18, experienced death during hospitalization, suffered from trauma or malignancy (including hematological malignancy), or were pregnant were excluded from the study. Following a comprehensive understanding and explanation of the study procedure, all participants provided written informed consent.

### Research variable

The following clinical data were collected within 24 h of admission: demographic information, clinical manifestations, comorbidities, various scoring systems upon admission, laboratory test results, etc. The follow-up indicators involved monitoring the post-discharge survival time of patients diagnosed with HRF for a period of 2 years. The patient’s tracking process was conducted by telephone interviews and the further verification of their condition was used by the hospital system.

The current project follows the principles of the Declaration of Helsinki. The study was approved by the ethics committees of the First People’s Hospital of Yancheng (No. 2020-K062) and the People’s Hospital of Jiangsu Province (No. 2021-SR-346). The participants at both hospitals were required to provide informed written consent in order to participate in the clinical study.

### Model construction and validation

**FIGURE 1:**
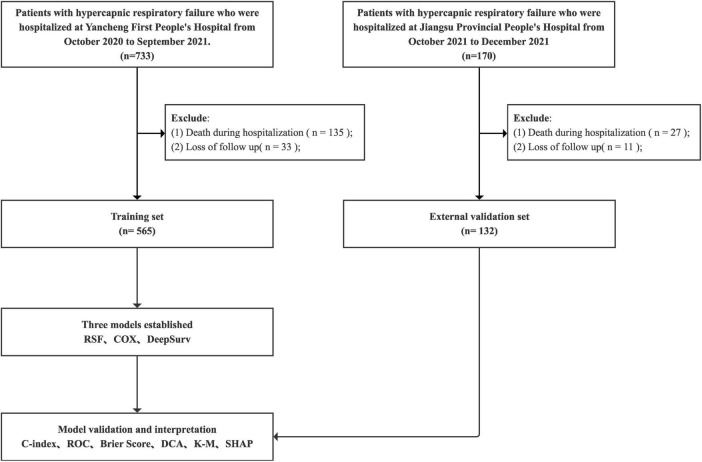
Flow chart of this study.

The modeling data was utilized for model construction and validation, while the external validation data was employed for subsequent model verification. The flowchart of this experiment for a multi-center prospective cohort study was depicted in Figure 1. Firstly, variable screening was performed using LASSO regression (Least Absolute Shrinkage and Selection Operator), followed by the construction of a multivariate CoxPH model based on the selected variables. Simultaneously, two models (RSF and DeepSurv) were constructed using the selected variables, and hyperparameters were optimized in the training set using mesh search method to determine the optimal values. The performance of the three models was subsequently assessed in both the modeling data set and the external validation data set.

CoxPH, as a classical parametric method, offers high interpretability and robustness. RSF employs non-parametric methods to capture nonlinear relationships and variable interactions, demonstrating greater resilience to data quality issues and multicollinearity. DeepSurv leverages deep learning techniques to handle high-dimensional and complex data, excelling in modeling nonlinear features and variable interactions. By integrating these three models, we can accommodate diverse data characteristics, achieve comprehensive and robust analysis both theoretically and practically, and provide multidimensional support for our research objectives.

The following evaluation indicators were employed: the conformance index (C-index) and the area under the receiver operating characteristic curve (at 6, 12, 18, and 24 months) was utilized to evaluate the discriminative ability of each model. The calibration of the model was assessed using Brier Score (at 6, 12, 18, and 24 months). Decision curve analysis (at 6, 12, 18, and 24 months) was utilized to calculate the clinical net benefit of each model.

The C-index reflects the ability of the model to distinguish between high and low risk individuals, with values ranging from 0.5 (nearly random) to 1.0 (completely accurate). The higher the value, the more accurately the model can distinguish between high and low risk individuals (17). The Brier Score assesses the accuracy and reliability of probabilistic predictions made by a model, with values ranging from 0 to 1. A lower score indicates a smaller prediction error, thereby reflecting greater accuracy and consistency in the model’s forecasts (18).

Time-dependent ROC analysis serves as a robust tool for assessing the predictive accuracy of survival models across various time points. By computing the sensitivity and specificity at each time point, a time-dependent ROC curve is generated, and the AUC (area under the curve) is calculated to quantify the model’s predictive performance. This method initially estimates an individual’s survival probability or risk score using a survival model. Subsequently, it constructs a time-dependent confusion matrix based on varying thresholds to evaluate the model’s classification performance at specific time intervals (19, 20). For right-censored data, the inverse probability weighting (IPW) method is employed to adjust for censoring bias, thereby ensuring the precision of AUC calculations. Through repeated analyses at multiple time points, the temporal changes in the model’s predictive power can be dynamically monitored and utilized for comparing different survival models (21, 22).

### Risk stratification, interpretation, and web-based prediction tool for optimal modeling

The risk score for each patient can be calculated using the optimal model derived from comparing various models. Patients were stratified based on their risk score, and survival analysis was conducted using Kaplan-Meier curves with differences compared using the log-rank test.

The SHAP plots served as a valuable tool for interpreting machine learning models. Within these plots, the length of the horizontal axis corresponding to each variable signified its contribution to the outcome, while the color of the dots represented the magnitude of that contribution.

The individual predictions comprised survival probability plots and local SHAP plots, which offered distinct perspectives on survival expectations and risk factors, respectively. The survival probability of each individual was calculated using non-parametric estimation. The local SHAP plot was an interpretive representation that showed the individual-specific contribution of a variable in a SHAP plot. The establishment of an association between risk factors and individual outcomes was facilitated based on the plots. Furthermore, we have developed a web-based prediction tool utilizing the Shiny framework to estimate patient survival probabilities at specified time points.

### Statistical analysis

The categorical variables in both the modeling data set and the external validation data set were presented using frequency and percentage, and analyzed using either chi-square test or Fisher exact test. Continuous variables conforming a normal distribution were represented by their mean and standard deviation, and analyzed using the *t*-test. Continuous variables not conforming to a normal distribution were represented by their median and quartile, and analyzed using the Mann-Whitney U test. A *p*-value < 0.05 in bilateral test was considered statistically significant. The mlr3proba package for R (version 0.4.13) was utilized to construct three survival machine learning models, wherein the DeepSurv model relied on the Pycox module. The survival machine learning model was explained using Python’s SHAP module (version 0.37.0). The CatPredi package in the R language was utilized for discretizing risk scores into layers.

## Results

### Clinical characteristics of patients with hypercapnic respiratory failure

The study included a total of 697 patients with HRF, and Table 1 presented the demographic and clinical information comparing the modeling data set to the external validation data set.

**TABLE 1:** The information for patients with hypercapnic respiratory failure in the training set and the external validation set.

Variables	Total (*n* = 697)	Training set (*n* = 565)	External validation set (*n* = 132)	*P*
Survival month	24.02 [7.39, 24.02]	24.02 [7.1, 24.02]	24.02 [8.72, 24.02]	0.151
Status				0.059
Alive	379 (54.38)	297 (52.57)	82 (62.12)	
Dead	318 (45.62)	268 (47.43)	50 (37.88)	
Treatment				0.385
Oxygen therapy	352 (50.5)	285 (50.44)	67 (50.76)	
Non-invasive ventilation	242 (34.72)	201 (35.58)	41 (31.06)	
Invasive and non-invasive sequential ventilation	103 (14.78)	79 (13.98)	24 (18.18)	
Gender				0.218
Female	246 (35.29)	206 (36.46)	40 (30.3)	
Male	451 (64.71)	359 (63.54)	92 (69.7)	
ICU admission				0.777
No	620 (88.95)	504 (89.2)	116 (87.88)	
Yes	77 (11.05)	61 (10.8)	16 (12.12)	
Smoking				< 0.001
No	297 (42.61)	222 (39.29)	75 (56.82)	
Yes	400 (57.39)	343 (60.71)	57 (43.18)	
Hypertension disease				0.499
No	422 (60.55)	346 (61.24)	76 (57.58)	
Yes	275 (39.45)	219 (38.76)	56 (42.42)	
Diabetes				0.999
No	593 (85.08)	481 (85.13)	112 (84.85)	
Yes	104 (14.92)	84 (14.87)	20 (15.15)	
Cerebrovascular				0.729
No	598 (85.8)	483 (85.49)	115 (87.12)	
Yes	99 (14.2)	82 (14.51)	17 (12.88)	
Cardiovascular				0.082
No	529 (75.9)	437 (77.35)	92 (69.7)	
Yes	168 (24.1)	128 (22.65)	40 (30.3)	
COPD				0.07
No	113 (16.21)	99 (17.52)	14 (10.61)	
Yes	584 (83.79)	466 (82.48)	118 (89.39)	
Asthma				0.999
No	681 (97.7)	552 (97.7)	129 (97.73)	
Yes	16 (2.3)	13 (2.3)	3 (2.27)	
ILD				0.999
No	679 (97.42)	550 (97.35)	129 (97.73)	
Yes	18 (2.58)	15 (2.65)	3 (2.27)	
Bronchiectasis				0.049
No	618 (88.67)	494 (87.43)	124 (93.94)	
Yes	79 (11.33)	71 (12.57)	8 (6.06)	
Pneumonia				< 0.001
No	532 (76.33)	451 (79.82)	81 (61.36)	
Yes	165 (23.67)	114 (20.18)	51 (38.64)	
Age	74 [68, 80]	74 [68, 80]	73.5 [67, 79.25]	0.959
BMI	21.8 [18.67, 25.39]	21.48 [18.38, 25.39]	22.6 [19.53, 25.71]	0.064
Fall risk score	4 [3, 5]	4 [3, 5]	4 [2.75, 5]	0.727
Braden score	18 [14, 20]	18 [15, 20]	17 [14, 20]	0.127
Barthel Index Rating Scale	56 [33, 76]	56 [35, 78]	58 [18, 68.75]	0.163
VAS pain score				0.949
0	618 (88.67)	501 (88.67)	117 (88.64)	
1	32 (4.59)	25 (4.42)	7 (5.3)	
2	36 (5.16)	30 (5.31)	6 (4.55)	
3	7 (1)	6 (1.06)	1 (0.76)	
4	4 (0.57)	3 (0.53)	1 (0.76)	
mMRC score				0.044
0	2 (0.29)	2 (0.35)	0 (0)	
1	22 (3.16)	13 (2.3)	9 (6.82)	
2	69 (9.9)	52 (9.2)	17 (12.88)	
3	211 (30.27)	170 (30.09)	41 (31.06)	
4	393 (56.38)	328 (58.05)	65 (49.24)	
Padua score	3 [1, 4]	3 [1, 4]	3 [1, 4]	0.73
PH	7.37 [7.31, 7.41]	7.37 [7.31, 7.41]	7.36 [7.32, 7.4]	0.666
Standard bicarbonate (mmol/L)	32.1 [29.1, 35.3]	32.2 [29.3, 35.6]	31.35 [28.78, 34.82]	0.334
PO2 (mmHg)	48 [40, 54]	49 [40, 56]	43 [37, 49]	< 0.001
PCO2 (mmHg)	65 [56, 78]	65 [56, 78]	64.5 [56, 77]	0.525
Blood gas calcium (mmol/L)	1.16 [1.12, 1.19]	1.16 [1.12, 1.2]	1.15 [1.1, 1.18]	0.007
Methemoglobin (%)	1.2 [1, 1.4]	1.2 [1, 1.4]	1.2 [1, 1.4]	0.385
Reduced hemoglobin (%)	4.6 [2.2, 9.1]	4.2 [2.2, 8.5]	6.2 [2.45, 11.43]	0.011
Hematocrit (%)	44 [38, 49]	43 [38, 49]	45 [39, 49]	0.228
Remaining alkaline (mmol/L)	9.4 [5.9, 13.5]	9.6 [6, 13.8]	8.7 [5.18, 13.3]	0.185
Lactic acid (mmol/L)	1.5 [1.2, 2]	1.5 [1.2, 1.9]	1.6 [1.2, 2.1]	0.063
Actual Bicarbonate (mmol/L)	37.8 [33.1, 42.8]	37.8 [33.3, 42.8]	37.8 [32.48, 42.55]	0.474
Carboxyhemoglobin (%)	2.4 [1.9, 2.9]	2.4 [1.9, 2.9]	2.3 [1.8, 2.82]	0.245
Oxyhemoglobin (%)	91.8 [87.2, 94.3]	92.2 [87.6, 94.4]	90.35 [85.15, 93.75]	0.015
Anion gap (mmol/L)	4 [2, 7]	4 [2, 7]	5 [1, 7]	0.982
TCO2 (mmol/L)	39.8 [35, 45.2]	39.8 [35.2, 45.4]	39.85 [34.2, 44.65]	0.464
Rdwcv (%)	13.5 [12.9, 14.6]	13.6 [12.9, 14.7]	13.4 [12.7, 14.5]	0.062
Rdwsd (fl)	46 [42.9, 49.8]	46.1 [43, 50.1]	45.65 [42.5, 48.85]	0.31
WBC (10^9^/L)	7.99 [6.2, 10.86]	7.82 [5.82, 10.47]	9.24 [6.84, 12.6]	< 0.001
Large platelet ratio (%)	34.6 [28.6, 43.1]	34.5 [28.6, 42.7]	34.95 [28.58, 43.78]	0.715
Monocytes percentage (%)	7.2 [4.8, 9.6]	7.2 [4.7, 9.4]	7.3 [4.8, 10.03]	0.477
Monocytes (%)	0.54 [0.38, 0.77]	0.53 [0.37, 0.75]	0.62 [0.44, 0.99]	0.001
Arterial hematocrit (%)	41 [36.2, 45.4]	40.8 [36, 45]	41.65 [37.1, 46.15]	0.079
RBC (10^12^/L)	4.44 [3.96, 4.93]	4.42 [3.95, 4.91]	4.61 [4, 4.95]	0.167
Lymphocytes percentage (%)	10.8 [5.5, 17.7]	11.3 [5.9, 17.9]	7.8 [4.3, 17.35]	0.01
Lymphocytes (10^9^/L)	0.84 [0.48, 1.26]	0.85 [0.5, 1.24]	0.74 [0.44, 1.34]	0.421
MCV (fl)	92.1 [88.3, 95.8]	91.9 [88.2, 96]	92.8 [88.6, 95.6]	0.34
MCH (pg)	30.1 [28.6, 31.3]	30.2 [28.6, 31.5]	29.8 [28.6, 30.83]	0.018
MCHC (g/L)	325 [315, 334]	326 [316, 336]	321 [312.75, 329]	< 0.001
MPV (%)	11.2 [10.5, 12.3]	11.2 [10.5, 12.2]	11.25 [10.5, 12.5]	0.635
Hemoglobin (g/L)	134 [118, 147]	134 [118, 147]	134 [118.75, 148.25]	0.646
PDW (fl)	13.8 [11.9, 16.2]	13.8 [11.8, 16.2]	13.65 [11.9, 16.62]	0.712
PLT (10^9^/L)	171 [127, 217]	168 [125, 215]	184 [143.25, 223]	0.038
PCT (%)	0.2 [0.15, 0.24]	0.19 [0.15, 0.24]	0.21 [0.17, 0.24]	0.008
Eutrophil percentage (%)	80 [70.9, 87.9]	79.7 [71.1, 87.5]	82.4 [70.45, 89.93]	0.235
Eutrophil (10^9^/L)	6.32 [4.47, 9.27]	6.19 [4.26, 8.97]	7.54 [5.38, 10.77]	< 0.001
D-dimer (mg/LFEU)	0.71 [0.38, 1.55]	0.69 [0.37, 1.53]	0.81 [0.42, 1.71]	0.214
APTT (S)	27.8 [25.6, 30.5]	27.8 [25.6, 30.6]	27.6 [25.7, 30]	0.611
Antithrombin III (%)	76.16 ± 15.27	75.65 ± 15.15	78.34 ± 15.65	0.075
TT (S)	15.8 [15, 17]	15.8 [15, 16.9]	16.25 [14.8, 17.52]	0.136
PT (S)	11.9 [11.2, 12.8]	11.8 [11.1, 12.7]	12.2 [11.4, 13.1]	0.003
FDP (ug/mL)	3.1 [2, 5.2]	3.1 [2.1, 5.1]	3 [1.9, 5.73]	0.574
FIB (S)	3.55 [2.65, 4.79]	3.51 [2.61, 4.58]	3.98 [2.91, 5.55]	0.004
GGT (U/L)	24.3 [17, 39]	24.8 [17, 39]	24 [18, 40.02]	0.845
Albumin (g/L)	35.88 ± 4.85	35.88 ± 4.75	35.87 ± 5.28	0.972
ALT (U/L)	26 [18, 39]	25 [16.6, 38]	31 [23.95, 44.25]	< 0.001
Cholinesterase (U/L)	5,000 [4,274, 6,062]	5,000 [4,319, 6,036]	5,093.5 [4,259.75, 6,366.25]	0.249
Serum calcium (mmol/L)	2.18 [2.09, 2.27]	2.18 [2.09, 2.26]	2.21 [2.1, 2.28]	0.323
Triglyceride (mmol/L)	0.98 [0.74, 1.35]	0.96 [0.73, 1.34]	1.06 [0.83, 1.44]	0.018
Creatinine (μmol/L)	65 [52.6, 83.2]	64.1 [51.7, 79.7]	69.25 [57.05, 88.6]	0.003
CK (U/L)	40 [30, 69]	38 [30, 66.4]	53 [34, 88.5]	< 0.001
CK_MB (U/L)	11.9 [9, 17]	11 [9, 16]	13 [10, 18]	0.013
AKP (U/L)	77.8 [66, 96]	77 [65, 95]	80.95 [66, 98.55]	0.316
Serum phosphorus (mmol/L)	1.22 [1.07, 1.41]	1.22 [1.06, 1.4]	1.21 [1.09, 1.44]	0.545
Urea nitrogen (mmol/L)	7.34 [5.28, 9.71]	7.29 [5.28, 9.4]	7.56 [5.39, 10.34]	0.293
Uric acid (μmol/L)	313.6 [231.7, 411.8]	310.1 [227, 406]	332.45 [252.8, 417.68]	0.249
Globulin (g/L)	28.5 [25.7, 31.7]	28.3 [25.6, 31.5]	29.15 [26.8, 31.92]	0.093
LDH (U/L)	338 [216, 497]	369 [224, 518]	258.5 [207.75, 432.25]	< 0.001
Serum bicarbonate (mmol/L)	35.9 [32, 39.2]	36.3 [32.6, 39.6]	32.95 [28.8, 37.38]	< 0.001
AST (U/L)	26 [20, 36]	26 [20, 35.8]	27 [21, 39.25]	0.216
Total cholesterol (mmol/L)	4.01 [3.34, 4.86]	4.01 [3.33, 4.83]	4.08 [3.39, 4.94]	0.543
Total bilirubin (mmol/L)	11.8 [8.3, 17]	11.9 [8.38, 16.89]	11.76 [8.16, 18.6]	0.853
Total protein (g/L)	64.76 ± 7.17	64.63 ± 7.11	65.31 ± 7.42	0.346
Myoglobin (ng/ml)	39.7 [29.2, 70.8]	40.3 [28, 68.5]	37.65 [30, 77.55]	0.404
NT-BNP (pg/ml)	538 [133, 2,580]	577 [130, 2,620]	412.5 [134.9, 2,310]	0.652

### LASSO regression screening variables

**FIGURE 2:**
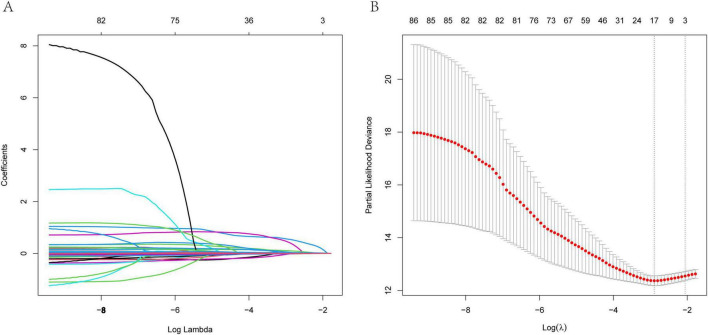
LASSO regression model was used to select the variables. **(A)** Path diagram of coefficient variation **(B)** A 10-fold cross-validation chart.

The study incorporated a total of 85 variables. Utilizing the training dataset, we employed the LASSO regression (Least Absolute Shrinkage and Selection Operator) method for variable selection. The optimal regularization parameter λ was determined through 10-fold cross-validation, with the model corresponding to the minimum value being selected as the final model (Figure 2). By incorporating an L1 norm regularization penalty term into the model, LASSO regression achieved both variable selection and coefficient shrinkage. This approach not only mitigated overfitting and enhanced the model’s generalization capability but also addressed multicollinearity issues. Through this method, 17 variables with the highest explanatory power for the research objective were ultimately selected, including: Gender, ICU admission, Hypertension disease, ILD, BMI, Braden score, mMRC score, Padua score, Carboxyhemoglobin, Arterial hematocrit, Lymphocytes percentage, Hemoglobin, GGT, Albumin, Cholinesterase, Urea nitrogen, LDH.

### Construction of three survival machine learning models

RSF model: The 17 variables selected by LASSO were used for modeling. Grid search was conducted, and a 10-fold cross-validation was performed to determine the optimal combination of hyperparameters for the model, including ntree set at 900, mtry set at 3, nodesize set at 18. The remaining hyperparameters were kept as default values.

CoxPH model: A multivariate Cox regression analysis was conducted using 17 variables selected by LASSO. The analysis identified ICU admission, ILD, BMI, mMRC score, Carboxyhemoglobin, and Urea nitrogen as significant prognostic factors in patients with HRF. The results of the multivariate Cox regression analysis were shown in Table 2.

**TABLE 2:** Univariate and multivariate Cox regression analysis for OS in training set.

Variables	HR	95% CI	*P*
Gender			
Female	Ref		
Male	1.198	0.934–1.538	0.156
ICU admission			
No	Ref		
Yes	1.998	1.404–2.845	< 0.001
Hypertension disease			
No	Ref		
Yes	0.933	0.725–1.202	0.594
ILD			
No	Ref		
Yes	2.717	1.536–4.806	0.001
BMI	0.957	0.931–0.984	0.002
Braden score	0.99	0.955–1.026	0.573
mMRC score	1.274	1.065–1.524	0.008
Padua score	1.067	0.998–1.141	0.057
Carboxyhemoglobin (%)	1.198	1.058–1.358	0.004
Arterial hematocrit (%)	0.974	0.919–1.031	0.364
Lymphocytes percentage (%)	0.987	0.972–1.003	0.112
Hemoglobin (g/L)	1.000	0.982–1.019	0.977
GGT (U/L)	1.000	0.998–1.003	0.639
Albumin (g/L)	0.992	0.966–1.018	0.534
Cholinesterase (U/L)	1.000	1.000–1.000	0.175
Urea nitrogen (mmol/L)	1.027	1.006–1.048	0.011
LDH (U/L)	1.000	1.000–1.000	0.776

DeepSurv model: The 17 variables selected by LASSO were used for modeling. Grid search and 10-fold cross-validation were used to determine the optimal combination of hyperparameters of the model, including alpha set at 0.5, learning_rate set at 0.005c, num_nodes set at 10. The remaining hyperparameters were kept as default values.

### Assessment and interpretation of the models

The C-index of each model was compared in both the modeling data set and the external validation set to compare their respective discriminatory abilities. The C indices of the RSF model, CoxPH model, and DeepSurv model (hereinafter referred to as the “three models”) on the modeling dataset were 0.792, 0.699, and 0.618, respectively. On the external validation set, these indices were 0.693, 0.681, and 0.532 for each respective model. These results indicated that the RSF model exhibited superior discriminative ability.

**FIGURE 3:**
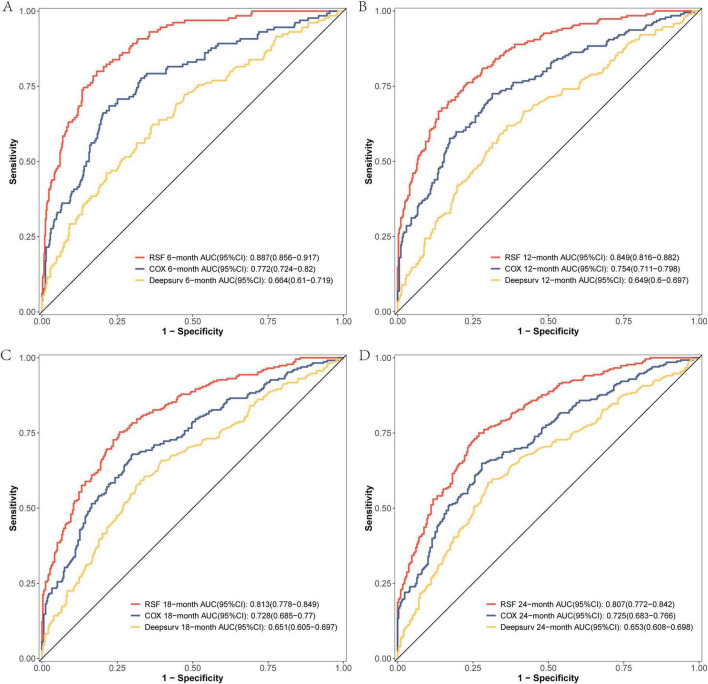
The ROC curves of the three models were plotted based on different time points in the training set: **(A)** at 6 months, **(B)** at 12 months, **(C)** at 18 months, and **(D)** at 24 months.

**FIGURE 4:**
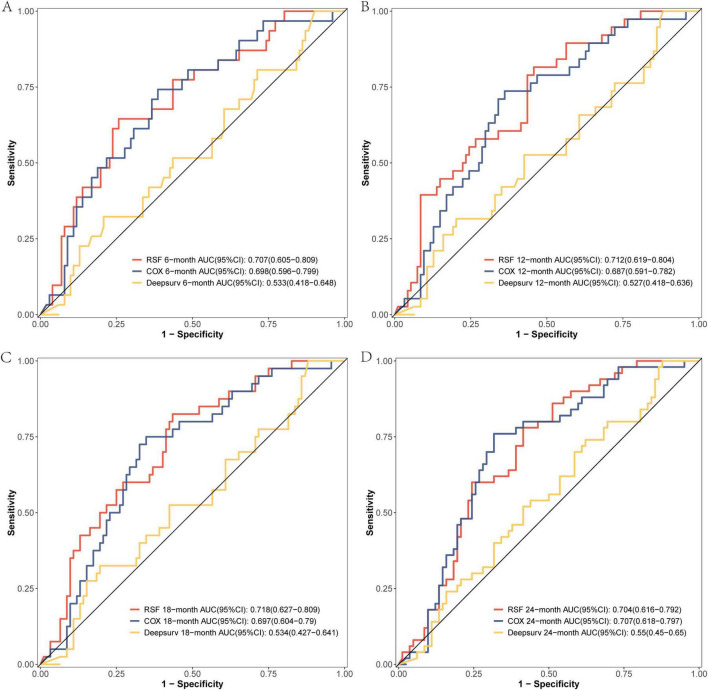
The ROC curves of the three models were plotted based on different time points in the External validation set: **(A)** at 6 months, **(B)** at 12 months, **(C)** at 18 months, and **(D)** at 24 months.

The comparison of the discrimination among the three models at 6, 12, 18, and 24 months on the modeling data set revealed that the RSF model exhibited superior discrimination (Figure 3). The external validation set demonstrated that the RSF model exhibited superior discrimination at 6, 12, and 18 months compared to the other models, while its discrimination at 24 months was comparable to that of the Cox regression model (Figure 4).

On the modeling data set, the Brier Score was utilized to assess the model’s predictive consistency at four time points: 6, 12, 18, and 24 months. The results showed that the Brier Score of the RSF model was the best and was less than 0.25 at each time point. Similarly, on the external validation set, the Brier Score was utilized to evaluate the model’s predictive consistency at 6, 12, 18, and 24 months. The results demonstrated that once again, the RSF model outperformed other models by maintaining a Brier Score below 0.25 for each respective time point. The above information was shown in Table 3.

**TABLE 3:** Information on the predictive accuracy of the model assessed at various time points.

Dataset	Model	Brier score			
		**6-month**	**12-month**	**18-month**	**24-month**
Training set	RSF	0.124	0.173	0.216	0.242
	COX	0.143	0.190	0.234	0.269
	DeepSurv	0.199	0.294	0.362	0.422
External validation set	RSF	0.164	0.188	0.203	0.221
	COX	0.175	0.206	0.218	0.229
	DeepSurv	0.259	0.267	0.261	0.277

**FIGURE 5:**
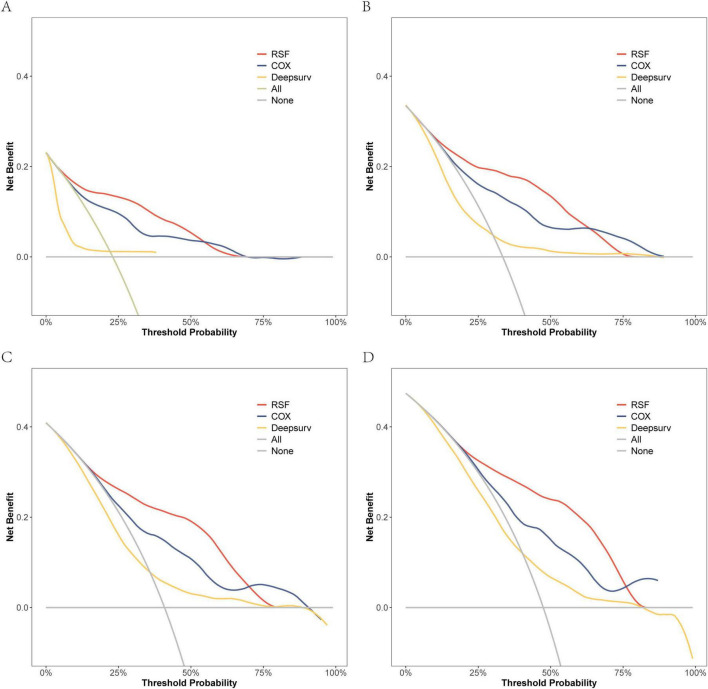
The DCA curves of the three models were plotted based on different time points in the training set: **(A)** at 6 months, **(B)** at 12 months, **(C)** at 18 months, and **(D)** at 24 months.

**FIGURE 6:**
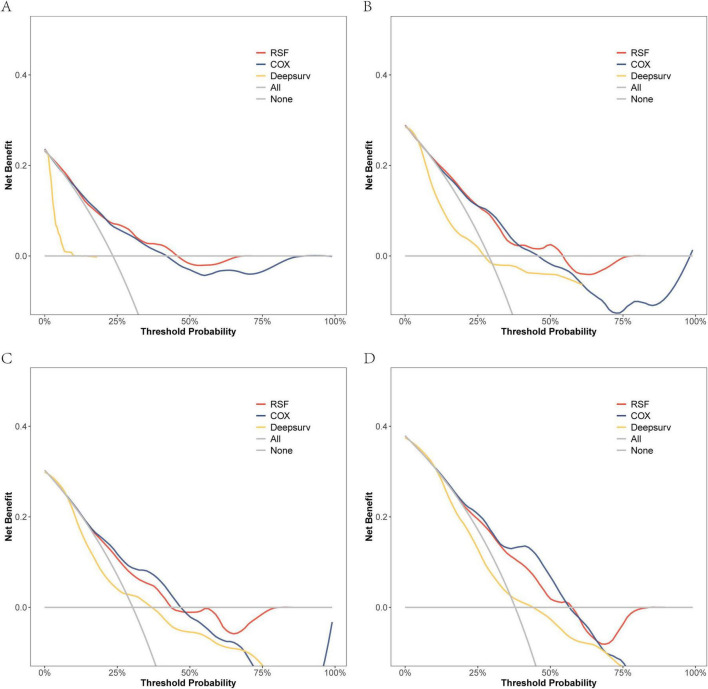
The DCA curves of the three models were plotted based on different time points in the External validation set: **(A)** at 6 months, **(B)** at 12 months, **(C)** at 18 months, and **(D)** at 24 months.

On the modeling data set, a DCA comparison was conducted for the three models at 6, 12, 18, and 24 months. The results revealed that the RSF model exhibited superior clinical net benefit (Figure 5). The RSF model also demonstrated superior clinical net benefit at 6, 12, and 18 months on the external validation set, while being comparable to Cox regression at 24 months (Figure 6).

In conclusion, the performance of the random survival forest (RSF) model in survival analysis demonstrated significant advantages. Specifically, regarding its differentiation ability, the C-index of the RSF model outperformed both the Cox regression model and the DeepSurv model across both the modeling data and external validation sets. This indicated that the RSF model possesses superior individualized prognostic differentiation capabilities. Regarding prediction consistency, the Brier Score of the RSF model demonstrated lower error rates across multiple time points, thereby further validating the accuracy and stability of its predictive performance. Simultaneously, clinical net benefit analysis demonstrated that the RSF model exhibited superior benefits during short-term follow-up and could more effectively support clinical decision-making. From a clinical perspective, the high predictive accuracy and interpretability of the RSF model not only enhance the precision of patient risk assessment but also provide a robust tool for personalized prognostic prediction and treatment optimization.

**FIGURE 7:**
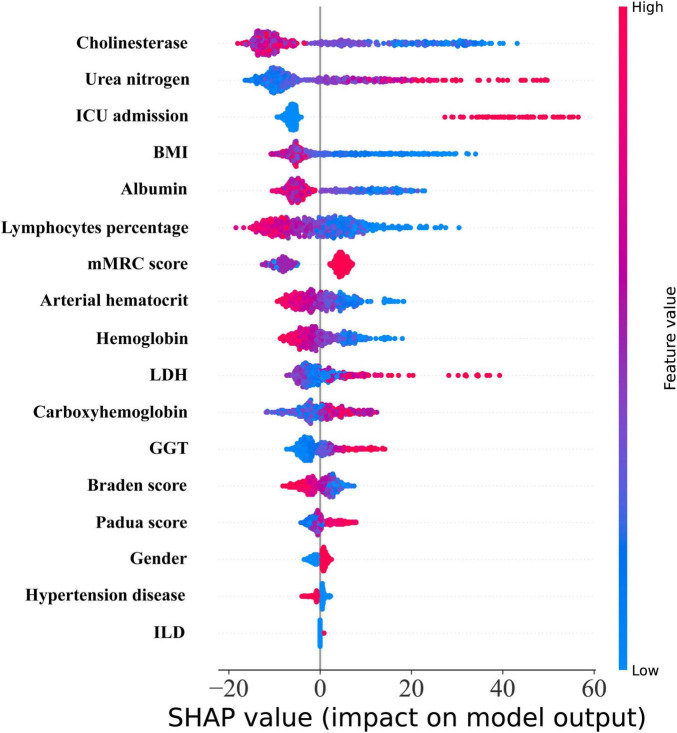
The SHAP diagram of RSF model.

Additionally, the RSF model was visually elucidated. In the SHAP diagram, the variables in the model were presented in a descending order of importance (Figure 7). Amongst the initial five variables, Cholinesterase emerged as the most crucial, succeeded by Urea nitrogen, ICU admission, BMI, and Albumin.

### Stratified risk of RSF in patients with hypercapnic respiratory failure

**FIGURE 8:**
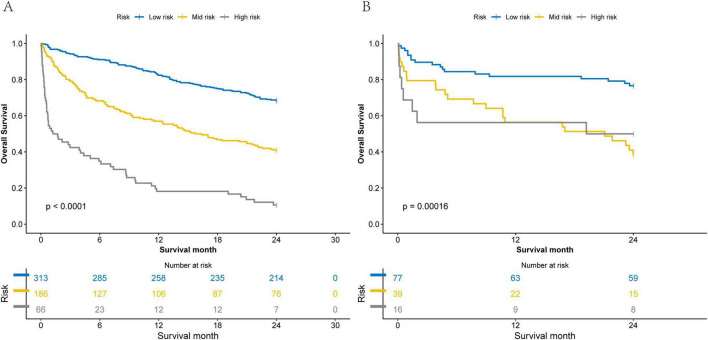
Risk stratification of the RSF model on the modeling data set **(A)** and the external validation data set **(B)**.

The patients in the validation set were categorized into three groups based on their risk scores: high-risk group (risk score > 1.06), medium-risk group (0.24 ≤ risk score ≤ 1.06), and low-risk group (risk score < 0.24). The Kaplan-Meier analysis and logarithmic rank test results depicted in Figure 8 demonstrated statistically significant differences among the high-risk, medium-risk, and low-risk groups as a whole.

### Clinical application of web-based prediction tools

The web-based prediction tool was developed utilizing the random survival forest (RSF) model. This tool, implemented via Shiny,^1^ offered a user-friendly interface, real-time predictive capabilities, and comprehensive visualization features. It served as an efficient and precise solution for individualized risk assessment and survival prediction, specifically tailored for clinical practitioners. By dynamically generating patient survival probability curves and estimating survival rates at specific time points, this tool offered robust quantitative support for clinical decision-making. It significantly enhanced the operability and adaptability of the model in practical applications, thereby providing crucial technical support for the individualized management of HRF.

### Prediction of individual prognosis in hypercapnic respiratory failure

**FIGURE 9:**
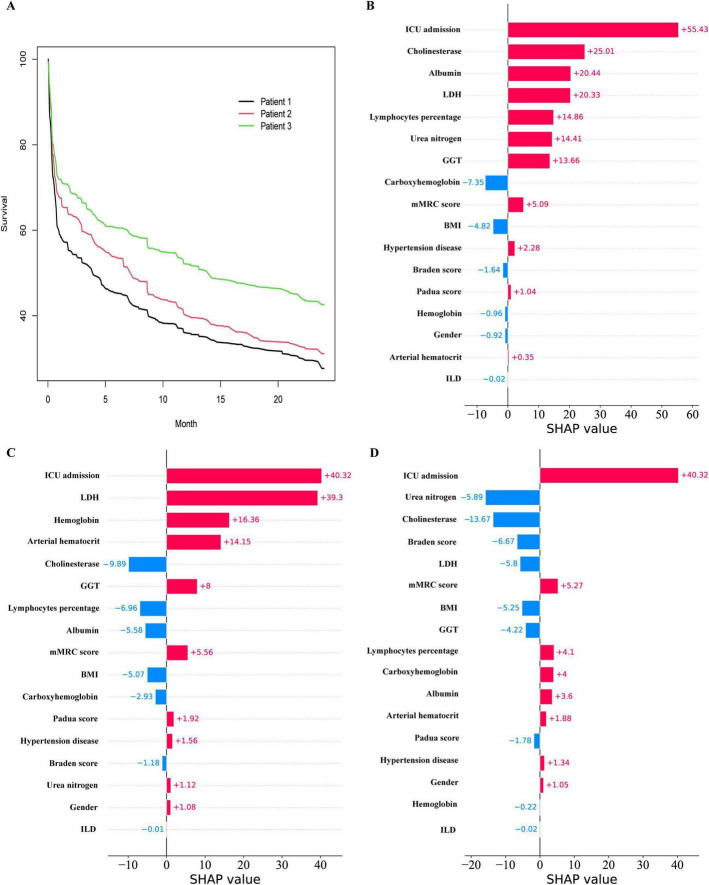
Prediction of Hypercapnic respiratory failure in individual patients. **(A)** the Survival curves for 3 patients. **(B)** the Local SHAP of patient 1. **(C)** the Local SHAP of patient 2. **(D)** the Local SHAP of patient 3.

Three patients were randomly selected and numbered sequentially to demonstrate individual prognosis. The individual predicted survival rate is illustrated in Figure 9A. It can be observed that the third patient exhibited a relatively favorable survival rate, whereas the first patient exhibited a relatively unfavorable survival rate. The local SHAP plot elucidated the prognosis for each patient in terms of the contribution of the variables, wherein red stripes represented risk factors associated with poor prognosis, while blue stripes indicated relative protective factors.

Patient 1: As shown in Figure 9B, the local SHAP plot revealed that the top five most important variables, namely ICU admission, Cholinesterase, Albumin, LDH, and Lymphocytes percentage, were all identified as risk factors associated with poor prognosis.

Patient 2: As shown in Figure 9C, the local SHAP plot revealed that ICU admission, LDH, Hemoglobin hematocrit and Arterial hematocrit were the risk factors with poor prognosis among the top five most important variables, while Cholinesterase was the protective factor.

Patient 3: As shown in Figure 9D, the local SHAP plot revealed that ICU admission was the risk factor with poor prognosis among the top five most important variables, while Urea nitrogen, Cholinesterase, Braden score and LDH were the protective factors.

## Discussion

Due to the high hospitalization rate and mortality in patients with HRF, it is necessary to carefully evaluate and select appropriate treatment to optimize the prognosis of patients (23). Previously, researchers have primarily focused on analyzing the risk factors influencing the survival of patients with HRF, but have not conducted modeling analyses to further understand these factors (4, 24–26). This is the inaugural clinical study that employs machine learning modeling for the prediction and management of HRF through the development, validation, and subsequent clinical implementation of the model. In this study, we developed three predictive models: the RSF, DeepSurv, and CoxPH models, and assessed their performance using metrics such as the C-index, ROC curve analysis, DCA, and Brier Score. The results indicated that the RSF model outperformed both the Cox regression model and the DeepSurv model in terms of modeling data and external validation data, albeit with some variations. Specifically, the C-index for the RSF model was 0.792 in the modeling dataset, which decreased to 0.693 in the external validation set. Despite this reduction, it still maintained superior discrimination. The Brier Score also exhibited a slight increase at various time points in the external validation data (e.g., from 0.124 to 0.164 at 6 months), indicating a modest decline in prediction consistency. Furthermore, Decision Curve Analysis (DCA) revealed that the RSF model’s external validation performance surpassed other models at 6, 12, and 18 months, although its advantage diminished and approached that of the Cox regression model at 24 months. These discrepancies may be attributed to differences in patient characteristic distributions, data heterogeneity, or uncertainties associated with extended follow-up periods. Future efforts should focus on collecting larger sample sizes or incorporating additional data dimensions to optimize the model, thereby enhancing its generalizability to external datasets.

Furthermore, the web-based prediction tool developed by our team utilizing the Shiny framework offers clinicians an advanced and reliable solution for personalized risk assessment and survival prediction. This tool featured an intuitive user interface, real-time predictive analytics, and visualization capabilities, enabling efficient and precise evaluation of patient outcomes. The tool was designed to dynamically generate patient survival probability curves and calculate survival rates at specific time points, thereby enhancing the clinical utility of the model. The tool facilitated data import, real-time computation, and multi-platform utilization, thereby enhancing its applicability in outpatient clinics, wards, and case discussions. Despite challenges related to data security and computational resources, these concerns were mitigated through on-premises hospital deployment, robust data encryption, and optimized computational algorithms. Consequently, this tool provided substantial support for precision medicine and personalized treatment strategies. Overall, this study offered valuable insights into modeling the prognosis of HRF. Furthermore, the web-based prediction tool developed by our team utilizing the Shiny framework offers clinicians advanced and reliable solutions for personalized risk assessment and survival prediction. This tool features an intuitive user interface, real-time predictive analytics, and visualization capabilities to efficiently and accurately assess patient outcomes. It is designed to dynamically generate patient-specific survival probability curves and calculate survival rates at designated time points, thereby enhancing the clinical utility of the model. The tool supports seamless data import, real-time computation, and multi-platform compatibility, improving its applicability in outpatient clinics, wards, and case discussions. Although challenges related to data security and computing resources exist, these concerns are addressed through on-premises hospital deployments, robust data encryption, and optimized computational algorithms. Consequently, the tool provides significant support for precision medicine and personalized treatment strategies. Overall, this study offers valuable insights into the prognosis of simulated HRF.

The RSF model surpasses traditional CoxPH models, particularly in the analysis of high-dimensional data, by automatically assessing intricate effects and interactions among all variables (27). Furthermore, by incorporating interpretative tools such as SHAP analysis, the critical variables within the RSF model and their contributions to the prediction outcomes can be systematically elucidated. For instance, the SHAP plot provides a visual representation of the positive and negative impacts of each variable on risk prediction, along with their relative significance. This aids clinicians in comprehending which factors are pivotal in influencing patient prognosis (28, 29). Simultaneously, local SHAP analysis facilitates personalized interpretation, allowing for the identification of the specific contributions of each patient’s unique variables to their respective risk scores (30). This interpretability not only bridges the gap between complex models and clinical applications but also equips physicians with a more robust decision-making foundation, thereby enhancing the acceptance and practical value of RSF models in precision medicine.

The difference is that DeepSurv model has been widely used in many survival analyses, has good predictive value, and its excellent performance is better than RSF model in many studies (31–33). However, the performance of the RSF model in this study surpasses that of the DeepSurv model for the following reasons: Firstly, the inherent “black box” nature of deep neural networks remains a hindrance (34); Secondly, it is possible that the size of the study data may not be sufficient to effectively train deep neural networks (35, 36).

In addition, most of the top five variables screened in the RSF model [Cholinesterase, Urea nitrogen (37, 38), ICU admission (3, 39), BMI (40, 41), and Albumin (42, 43)] have been shown to be associated with death or survival prognosis in patients with HRF. However, Cholinesterase has not been linked to HRF, which is a novel and significant finding in our study and associated with the survival prognosis in patients with HRF. The specific reason may be that serum Cholinesterase is correlated with heightened inflammation, escalated disease severity, and deteriorating prognosis in critically ill patients (44). The serum cholinesterase activity has also been linked to adverse outcomes in critically ill patients who are admitted to the ICU due to acute respiratory failure following COVID-19 infection (45).

Therefore, recognizing and enhancing awareness of these risk factors is crucial for early intervention and appropriate treatment of patients with HRF. Precise survival predictions offer a more reliable individualized prognosis assessment, thereby minimizing unnecessary medical interventions and cost inefficiencies. Consequently, the application of the random survival forest (RSF) model in clinical practice holds significant potential, particularly in supporting clinical decision-making. The model can be integrated into electronic medical record systems to provide personalized survival predictions and risk stratification by incorporating key clinical indicators of patients. This integration helps optimize resource allocation and improve treatment efficiency, especially in resource-constrained settings or varying medical conditions. However, the broad application of the model faces certain limitations, such as data discrepancies between different medical institutions that may reduce the model’s generalization ability. Therefore, considering the variations in patient characteristics and data quality across different settings, the model may require appropriate adjustments in practical applications to ensure its reliability and applicability. Additionally, future studies should further validate the model’s performance across multiple regions, institutions, and diverse patient populations. Efforts should also focus on optimizing model performance and exploring methods for dynamic survival prediction and cloud deployment to support a wider range of clinical scenarios.

The study is subject to several limitations. First, additional multi-center data sets are required to assess the stability and validity of the model. Second, although machine learning methods exhibit advantages in handling limited sample sizes, their predictive power must be validated through replication in a broader population. Additionally, the current study did not delve into the effects of different patient subgroups on model performance, and further refinement of subgroup analysis is necessary to enhance the model’s applicability (46). Third, our developed model solely utilizes clinical variables; however, other factors such as medical imaging and omics data may possess clinical significance in predicting HRF. Finally, it is important to note that the prediction model is based on the Chinese population; therefore, further verification is necessary to determine its applicability to other ethnic groups.

In summary, a machine learning model was developed utilizing clinical variables to accurately predict survival prognosis in patients with HRF. The RSF model may offer distinct advantages over both the CoxPH model and the DeepSurv model, making it a valuable tool for clinical evaluation and patient monitoring.

## Data Availability

The original contributions presented in this study are included in this article/supplementary material, further inquiries can be directed to the corresponding author.

## Footnotes

1
https://app-predict.shinyapps.io/rsf-model/
